# Die Hand positionieren, um zu agieren – Validierung des 8-Punkte-Greifraum-Tests

**DOI:** 10.1007/s00391-022-02029-3

**Published:** 2022-02-24

**Authors:** Sonja Krupp, Theresa Petersen, Friedrich Balck, Meike Kasten, Martin Willkomm, Jennifer Kasper

**Affiliations:** 1 Forschungsgruppe Geriatrie Lübeck, Krankenhaus Rotes Kreuz Lübeck – Geriatriezentrum, Marlistr. 10, 23566 Lübeck, Deutschland; 2 Abteilung Psychosoziale Medizin und Entwicklungsneurowissenschaften, Med. Fakultät Carl Gustav Carus, Technische Universität Dresden, Dresden, Deutschland; 3 Klinik für Psychiatrie und Psychotherapie, Universität zu Lübeck, Lübeck, Deutschland

**Keywords:** Geriatrisches Assessment, Obere Extremität, Handkraft, Motorik, Gelenkbeweglichkeit, Geriatric assessment, Upper extremity, Hand strength, Motor skills, Joint mobility

## Abstract

**Hintergrund:**

Das standardisierte geriatrische Assessment der oberen Extremitäten beschränkt sich häufig auf die Messung der Handkraft. Als einziges weiteres Instrument nennt die S1-Leitlinie zum geriatrischen Assessment der Stufe 2 den 20-Cents-Test (20-C-T). Neben Kraft und Feinmotorik ist jedoch das erfolgreiche Platzieren der Hand eine Voraussetzung für die Selbstversorgung.

**Ziel der Arbeit:**

Zur standardisierten seitengetrennten Überprüfung des alltagsrelevanten Positionierens der Hand im Sitzen wurde der 8‑Punkte-Greifraum-Test (8P-GRT) entwickelt. Die Studie diente der Ermittlung von Gütekriterien des 8P-GRT bei geriatrischen Krankenhauspatienten.

**Material und Methoden:**

Zwischen dem 31.07.2019 und dem 23.09.2019 wurden im Krankenhaus Rotes Kreuz Lübeck – Geriatriezentrum 82 stationäre Patienten mithilfe des 8P-GRT, des Shoulder Pain and Disability Index (SPADI), einem zu den Handpositionen des 8P-GRT korrespondierenden Fragebogen zu Aktivitäten der Selbstversorgung, der Handkraftmessung und dem 20-C‑T untersucht.

**Ergebnisse:**

Die Interrater-Reliabilität betrug 0,99, die Retest-Reliabilität 0,95 für die rechte und 0,90 für die linke Seite. Auf die Person bezogen, trat ein Deckeneffekt (beidseits Score 8) bei 4,1 % (*n* = 3) auf; kein Bodeneffekt wurde beobachtet. Die interne Konsistenz (Cronbachs α) des gemäß Faktorenanalyse zweifaktoriellen Tests betrug 0,78 (rechts) bzw. 0,76 (links). Jeder der anderen Tests korrelierte enger mit dem 8P-GRT auf der rechten Seite, wobei die Korrelation mit dem oben genannten Fragebogen am höchsten war (−0,72), gefolgt vom SPADI (−0,60).

**Diskussion:**

Eine standardisierte Erhebung der Handkraft, Feinmotorik und aktiven Positionierung der Hand (z. B. 8P-GRT) fügt die Hauptaspekte der Funktionsfähigkeit der oberen Extremitäten zu einem Gesamtbild zusammen.

## Abstract (en)

**Background:**

The standardized geriatric assessment of the upper extremities is often limited to measurement of hand strength. The only other instrument mentioned in the S1 guidelines on level 2 geriatric assessment is the 20 cents test (20-C-T); however, in addition to strength and fine motor skills, successful hand placement is a prerequisite for self-care.

**Objective:**

The 8‑point reaching range test (8P-GRT) was developed for standardized separate testing of sides in a seated person concerning hand positioning relevant to daily living. The purpose of the study was to determine quality criteria of the 8P-GRT in geriatric hospital patients.

**Material and methods:**

Between 31 July 2019 and 23 September 2019, a total of 82 inpatients were examined at the Hospital Red Cross Lübeck Geriatrics Center using the 8P-GRT, shoulder pain and disability index (SPADI), a questionnaire on self-care activities corresponding to the hand positions of the 8P-GRT, hand strength measurement and the 20-C‑T.

**Results:**

The interrater reliability was 0.99 and the retest reliability was 0.95 for the right side and 0.90 for the left side. On the individual level a ceiling effect (both sides score 8) occurred in 4.1% (*n* = 3) but no floor effect was observed. The internal consistency (Cronbachʼs alpha) of the two-factorial test according to factor analysis was 0.78 (right) and 0.76 (left). Each of the other tests correlated more closely with the 8P-GRT on the right side, whereby the correlation was highest with the abovenamed questionnaire (−0.72), followed by the SPADI (−0.60).

**Conclusion:**

A standardized survey of hand strength, fine motor skills and active positioning of the hand (e.g., 8P-GRT) synthesizes the main aspects of upper extremity functioning into an overall picture.

*Hand*lungsfähigkeit ist eng mit den oberen Extremitäten verbunden. In das standardisierte geriatrische Assessment dieser Region haben im deutschsprachigen Raum lediglich die Hand- oder Greifkraft [1] und in geringerem Umfang der 20-Cents-Test (20-C-T) [2] Eingang gefunden. Diese Parameter werden in der S1-Leitlinie „Geriatrisches Assessment der Stufe 2 – Living Guideline“ [3] erwähnt. Für die Alltagsbewältigung ist jedoch auch das erfolgreiche Positionieren der Hand eine Voraussetzung. Diese Fähigkeit kann mithilfe des 8‑Punkte-Greifraum-Tests (8P-GRT) überprüft werden, dessen Validierung der Beitrag vorstellt.

## Hintergrund

Die Selbstversorgung eines Menschen ist in hohem Maße vom erfolgreichen Einsatz seiner Hände abhängig. Neben Kognition und Sensorik spielen die Muskelkraft und koordinative Fähigkeiten wie die Feinmotorik eine Rolle. Der Einsatz der Hand ist jedoch auch limitiert, wenn sie nicht dort platziert werden kann, wo sie tätig werden soll. Insbesondere die aktive Beweglichkeit des Schultergelenks ist im Alter häufig alltagsrelevant beeinträchtigt. Über die Hälfte komplexer Humerusfrakturen betrifft Frauen im Alter ab 60 Jahren [4]. Die Prävalenz symptomatischer Rupturen der Rotatorenmanschette beträgt im 8. Lebensjahrzehnt 50 % [5]; auch Impingement-Syndrom und Arthrose gehören im Alter zu den häufigen, den Greifraum potenziell reduzierenden Erkrankungen. In der Orthopädie gebräuchliche Verfahren wie z. B. der Schulterfunktion-Score nach Constant und Murley [6] sprengen vom Aufwand her den Umfang, der im Rahmen des geriatrischen Assessments der Stufe 2 leistbar ist. Außerdem ist der Greifraum nicht allein von einer intakten Schulterfunktion abhängig, sondern wird auch durch Hand‑, Ellenbogen‑, Wirbelsäulen- und Hüftbeweglichkeit sowie die Balance beeinflusst.

Für Personen, die sich nicht selbstständig fortbewegen können, ist der Greifraum besonders bedeutsam, da er ihren Aktionsradius darstellt. Im Assessment der Beweglichkeit der Bewohner in Pflegeeinrichtungen hat die Forschungsgruppe Geriatrie Lübeck sich daher auf den Greifraum fokussiert. In Ermangelung eines geeigneten Performance-Tests, der seitengetrennt auch für kognitiv eingeschränkte ältere Menschen mit einer Dauer unter 5 min im Sitzen durchführbar ist, wurde 2015 der 8‑Punkte-Greifraum-Test (8P-GRT) entwickelt und zur Effektevaluation eingesetzt [7]. Bei den 220 mithilfe des 8P-GRT untersuchten pflegebedürftigen Senioren betrug der daraus gebildete Score im Mittel rechts 5,84 ± 1,86 und links 5,96 ± 1,95 Punkte; die Kognition der Senioren hatte keinen signifikanten Einfluss auf das Testergebnis. Eine Validierungsstudie mit geriatrischen Krankenhauspatienten sollte klären, ob der 8P-GRT die Voraussetzungen dazu erfüllt, die Selbstversorgung beeinträchtigende Einschränkungen des Greifraums standardisiert zu erfassen, um sie in der Therapie zu berücksichtigen.

## 8-Punkte-Greifraum-Test

Orientiert an im Barthel-Index [8] erfassten Aktivitäten (Nahrungsaufnahme, Körperpflege, einschließlich Toilettengang, An- und Ausziehen) wurden 8 Handpositionen so gewählt, dass ihr Erreichen den Greifraum repräsentiert, der für die Selbstversorgung einer sitzenden Person relevant ist. Die Reihenfolge der Durchführung ist frei wählbar, ebenso, ob beide Seiten abwechselnd (dies erleichtert kognitiv beeinträchtigten Personen das Verständnis) oder nacheinander (dies ermöglicht eine fließende Bewegung Kinn – Scheitel – Nacken – Elevation – Boden – Steiß – Lumbalwirbel – zwischen die Scapulae) getestet werden. Die 8 Aufgabenstellungen in wörtlicher Rede in kursiver Schrift enthält Tab. 1; in Klammern finden sich zusätzliche Hinweise zur Bewertung. Diese erfolgt binär (1: erreicht, 0: nicht erreicht). Der durch Addition gewonnene Score wird für jede Seite getrennt angegeben. Die Tabelle nennt ebenfalls für jedes Item repräsentative Aktivitäten der Selbstversorgung.

**Table 1:** 

Items des 8P-GRT	Rechts	Links	Beispiele für Aktivitäten
*„Bitte berühren Sie mit Ihrer Handfläche das Kinn.“* (Handfläche ≙ Handteller)	0/1	0/1	Nahrung zum Mund führen, Rasieren, Zahnprothese einsetzen
*„Bitte legen Sie Ihre Handfläche auf Ihren Kopf.“* (Auf den höchsten Punkt)	0/1	0/1	Kämmen, Mütze aufsetzen
*„Bitte umfassen Sie mit Ihrer Hand Ihren Nacken.“* (Wirbelsäule gerade halten, Grundgelenke des 2. bis 5. Fingers erreichen Wirbelsäule)	0/1	0/1	Haare und Nacken waschen
*„Bitte heben Sie Ihren Arm gestreckt so hoch wie möglich.“* (Oberarm mindestens 150°-Anteversion)	0/1	0/1	Erhöht stehende Objekte nehmen, z. B. für die Köperpflege im Bad
*„Bitte berühren Sie mit den Fingerspitzen den Boden direkt neben Ihrem Schuh.“* (Egal, an welcher Seite des Schuhs)	0/1	0/1	Etwas vom Boden aufheben, Socken und Schuhe anziehen, Füße abtrocknen
*„Bitte schieben Sie Ihre Hand mittig unter Ihren Po.“* (Finger auf der Sitzfläche, Handteller auf dem Kreuzbein)	0/1	0/1	Sich nach dem Stuhlgang säubern
*„Bitte legen Sie Ihre Hand hinten auf den Hosenbund/unteren Lendenwirbel.“* (Egal, ob mit dem Handteller oder Handrücken)	0/1	0/1	Unteren Rücken waschen, Hose hochziehen, Hemd in die Hose stecken
*„Bitte greifen Sie mit den Fingern zwischen die Schulterblätter.“* (Mindestens 2 Finger erreichen die Linie zwischen den unteren Schulterblattspitzen)	0/1	0/1	Oberen Rücken waschen oder eincremen, Büstenhalter schließen
**Summenscore**	(0–8)	(0–8)	–

## Studiendesign und Methoden

Die Validierung des 8P-GRT sollte im Rahmen einer monozentrischen Kohortenstudie an akutstationären geriatrischen Patienten erfolgen, die folgende Einschlusskriterien erfüllen:

- beide Arme ohne Verbände, Orthesen oder vorgeschriebene Bewegungslimitation,
- kein Hinweis auf übertragbare Keimbesiedlung oder Infektion,
- Sitzstabilität von mindestens 30 min,
- ausreichende Kommunikationsfähigkeit, um die vorgesehenen Fragebogen voraussichtlich zu verstehen (ausreichende Seh- und Hörfähigkeit sowie Deutschkenntnisse, kein Hinweis auf höhergradige kognitive Störung gemäß ärztlichem Urteil) und
- schriftliche Einwilligung zur Studienteilnahme.

Für die Datenanalyse wurde SPSS Version 28 (IBM SPSS Statistics for Windows, Armonk, NY, USA) eingesetzt. Die Stichprobenbeschreibung erfolgte durch Mittelwert, Standardabweichung und Streuung. Das Signifikanzniveau wurde auf α = 0,05 festgesetzt. Für die Retest- sowie Interrater-Reliabilität wurde der 8P-GRT nach einem bis 3 Tagen erneut durchgeführt, diesmal von 2 Untersuchenden beobachtet und bewertet. Reliabilität, konvergente und divergente Validität wurden mithilfe der Korrelation nach Spearman berechnet, die interne Konsistenz mit Cronbachs α angegeben. Eine Faktorenanalyse erfolgte nach dem Hauptkomponentenmodell unter Beachtung des Kaiser-Kriteriums.

Für die Studie liegt ein positives Votum der Ethikkommission der Universität zu Lübeck (Aktenzeichen 19-220) vor.

### Shoulder Pain and Disability Index

Aus den in der Literatur beschriebenen Instrumenten kam kein Performance-Test als Vergleichsstandard für die konvergente Validität in Betracht. Am ehesten geeignet erschien der Shoulder Pain and Disability Index (SPADI; [9, 10]), eine Selbstbeurteilungsskala mit validierter deutscher Übersetzung [11], deren Subskala für Alltagsaktivitäten gesondert anwendbar ist [12]. Die Bewertung der Schwierigkeiten, sich (1.) die Haare und (2.) den Rücken zu waschen, (3.) Pulli bzw. (4.) Hemd/Bluse (vorn geknöpft) und (5.) Hose anzuziehen, (6.) einen Gegenstand auf ein hohes Regal zu legen, (7.) 5 kg zu tragen und (8.) etwas aus der hinteren Hosentasche zu nehmen, erfolgt zwischen 0 (ohne Schwierigkeit durchführbar) bis 10 Punkten (Durchführung unmöglich). Der Quotient aus Summenscore und dem Maximalscore 80 wird in Prozent angegeben.

In der Studie wurde bei der Angabe von Beeinträchtigungen zusätzlich deren Ursache aus Patientensicht erfragt (Fingerfertigkeit, Handkraft, Beweglichkeit, anderes).

### Fragebogen zu mit dem Greifraum assoziierten Selbstversorgungsaktivitäten

Da die im SPADI erfragten Tätigkeiten nicht sämtlich denen des 8P-GRT entsprachen, wurde für die Inhaltsvalidität ein Fragebogen (Tab. 2) konstruiert, auf dem die Patienten Schwierigkeiten bei der Ausführung von zu den Handpositionen korrespondierenden Aktivitäten der Selbstversorgung 3‑stufig bewerten sollten. Zusätzlich wurde nach dem Vorliegen weiterer Beeinträchtigungen durch eingeschränkte Beweglichkeit gefragt.

**Table 2:** 

Seitengetrennte Bewertung mit: „Ja, ohne Schwierigkeiten“ (0); „Ja, mit Schwierigkeiten“ (1); „nein“ (2)	Punkte
*„Reicht Ihre Beweglichkeit, *	
*… um sich Essen zum Mund zu führen?“*	0/1/2
*… um sich zu kämmen?“*	0/1/2
*… um sich die Haare zu waschen?“*	0/1/2
*… um etwas von einem Regalbrett zu nehmen, das höher als ihr Kopf ist?“*	0/1/2
*… um etwas vom Boden aufzuheben, was neben Ihrem Schuh liegt?“*	0/1/2
*… um sich nach dem Stuhlgang zu säubern?“*	0/1/2
*… um sich den unteren Rücken zu waschen und sich hinten das Hemd in die Hose/den Rock zu stecken?“*	0/1/2
*… um sich ggf. den BH hinten zu schließen oder sich in diesem Bereich den oberen Rücken einzucremen?“*	0/1/2
Summe	0–16

### Diskriminante Validität: Handkraftmessung und 20-Cents-Test

Die Messung der Hand- oder Greifkraft [1] erfolgte beidseits alternierend je 3‑mal mithilfe eines Dynamometers (Kern MAP Handkraftmesser 40K1). Dabei hielt der sitzende Patient das Gerät in der auf dem ipsilateralen Oberschenkel liegenden Hand. Der höchste Wert einer Seite wurde für die Datenanalyse verwendet.

Für die Feinmotorik wurde mithilfe einer Stoppuhr erhoben, wie viel Zeit benötigt wurde, um 20 auf Zeichenblockpapier ausliegende 1‑Cent-Münzen einzeln mit den Fingerspitzen einer Hand in ein Auffanggefäß zu transportieren [2]. Die maximale Testdauer beträgt 60 s. Für die Datenanalyse wird der Quotient Cents/s verwendet.

## Ergebnisse

Zwischen dem 31.07.2019 und dem 23.09.2019 wurden im Krankenhaus Rotes Kreuz Lübeck – Geriatriezentrum 282 Akten aktuell stationärer Patienten auf das Vorliegen der Einschlusskriterien gesichtet. Die häufigsten Ausschlusskriterien waren eine Demenz (*n* = 26, 38 %) oder angeordnete Bewegungslimitierungen im Bereich der oberen Extremitäten (*n* = 13, 19 %). Von 214 infrage kommenden Patienten wurden 112 ohne Hinweis auf einen eingeschränkten Greifraum nicht um Teilnahme gebeten, um den Anteil an beeinträchtigten Probanden in der Stichprobe zu erhöhen.

Von den verbleibenden 102 Patienten stimmten 82 (51 Frauen, 62 %; 31 Männer, 38 %) im durchschnittlichen Alter von 81,6 ± 6,4 Jahren (Range 64 bis 96 Jahre) der Teilnahme zu. Der Mittelwert des Barthel-Index bei Aufnahme betrug 51,8 ± 14,2 (Range 10–85).

Der Zeitaufwand für die Durchführung des 8P-GRT betrug für beide Seiten insgesamt im Mittel 136 ± 43 s (Range 54–235 s), für den sich auf die korrespondierenden Aktivitäten beziehenden Fragebogen 189 ± 85 s (Range 70–440 s). Die Häufigkeitsverteilung des 8P-GRT-Scores (Anzahl erfolgreicher Handpositionierungen) zeigt Tab. 3. Auf die Person bezogen wurde der Bodeneffekt bei keinem, der Deckeneffekt (beidseits Score 8) bei 3 Probanden (4,1 %) erreicht.

**Table 3:** 

Punkte	Rechts, *n* (%)	Links, *n* (%)
0	2 (2)	3 (4)
1	4 (5)	1 (1)
2	6 (7)	5 (6)
3	8 (10)	1 (1)
4	8 (10)	5 (6)
5	11 (13)	12 (15)
6	14 (17)	23 (28)
7	21 (26)	21 (26)
8	8 (10)	11 (13)
Summe	82 (100)	82 (100)

In Tab. 4 sind die Items des 8P-GRT anhand der errechneten Mittelwerte nach Schwierigkeit aufsteigend angeordnet. Geschlecht, Alter, Seite und faktische Händigkeit (subjektiv geschicktere Hand) hatten keinen signifikanten Einfluss auf den Summenscore. Die Retest-Reliabilität war mit rechts 0,95 (0,91–0,98; *p* = 0,000) und links 0,90 (0,81–0,96; *p* = 0,000) hoch, die Interrater-Reliabilität mit 0,99 beidseits (rechts 0,99–1,00, links 0,98–1,00; jeweils *p* = 0,000) noch höher, überprüft an jeweils 81 Patienten.

**Table 4:** 

Aufgabenformulierung	Re. Hand MW ± SD	Li. Hand MW ± SD
*„Bitte berühren Sie mit Ihrer Handfläche das Kinn.“*	0,98 ± 0,16	0,96 ± 0,19
*„Bitte legen Sie Ihre Hand hinten auf den Hosenbund/unteren Lendenwirbel.“*	0,78 ± 0,42	0,89 ± 0,32
*„Bitte legen Sie Ihre Handfläche auf Ihren Kopf.“*	0,74 ± 0,44	0,85 ± 0,36
*„Bitte heben Sie Ihren Arm gestreckt so hoch wie möglich.“*	0,70 ± 0,46	0,80 ± 0,40
*„Bitte schieben Sie Ihre Hand mittig unter Ihren Po.“*	0,68 ± 0,47	0,74 ± 0,44
*„Bitte umfassen Sie mit Ihrer Hand Ihren Nacken.“*	0,62 ± 0,49	0,70 ± 0,46
*„Bitte berühren Sie mit den Fingerspitzen den Boden direkt neben Ihrem Schuh.“*	0,42 ± 0,50	0,49 ± 0,50
*„Bitte greifen Sie mit den Fingern zwischen die Schulterblätter.“*	0,18 ± 0,39	0,26 ± 0,44
Summenscore		
Frauen	4,94 ± 2,30	5,71 ± 2,00
Männer	5,48 ± 1,80	5,68 ± 1,85

*MW* Mittelwert, *SD* Standardabweichung, *re* rechte, *li* linke

Cronbachs α als Maß der internen Konsistenz des 8P-GRT betrug 0,78 (rechts) bzw. 0,76 (links). In der für die Bestimmung der Konstruktvalidität durchgeführten Faktorenanalyse fanden sich 2 Faktoren mit Eigenwerten über 1 (rechts 3,33 und 1,15, links 3,45 und 1,02) nach dem Kaiser-Kriterium; diese erklärten zusammen 56 % der Varianz. Die Varimax-rotierte Matrix ließ nicht über die Faktorladungen der Items erkennen, welche Inhalte die Faktoren repräsentieren; der dominierende Faktor entspricht am ehesten der Anteversion/Elevation des Arms.

Die Korrelation nach Spearman zwischen den beiden Fragebogen und dem 8P-GRT war bezüglich der rechten, nahezu stets als faktisch dominant angegebenen Seite höher als bezüglich der linken Seite (Tab. 5). In den Fragebogen genannte Defizite wurden von den Probanden teilweise einer gestörten Feinmotorik, Schwindel oder mangelnder Sicherheit und Ausdauer beim Stehen zugeordnet statt verminderter Beweglichkeit.

**Table 5:** 

Instrument	*r*		95 %-Konfidenzintervall		*p*	
	Rechts	Links	Rechts	Links	Rechts	Links
Fragebogen zu 8 Alltagsaktivitäten	−0,72	−0,53	−0,83–−0,57	−0,70–−0,32	0,000	0,000
SPADI	−0,60	−0,36	−0,76–−0,40	−0,57–−0,11	0,000	0,007
Handkraft	0,39	0,28	0,20–0,57	0,06–0,49	0,000	0,011
20-Cents-Test	0,40	0,10	0,20–0,57	−0,10–0,36	0,000	0,235

*SPADI* Shoulder Pain and Disability Index

## Diskussion

Aufdecken und Präzisieren von Therapiebedarf sowie Monitoring des Verlaufs – Maßnahmen, die in Bezug auf die unteren Extremitäten selbstverständlich sind, sollten ebenso für die oberen Extremitäten gelten. Hierzu sollte neben der Kraft und Feinmotorik auch die alltagsrelevante aktive Beweglichkeit überprüft werden.

Gegenüber dem SPADI und anderen Fragebogen, die auf die Selbstbeurteilung von Fähigkeiten abzielen, bietet der 8P-GRT folgende Vorteile:

**1**. Der 8P-GRT ist – wie sich in Untersuchungen in Pflegeeinrichtungen zeigte – auch bei kognitiv beeinträchtigten Personen einsetzbar, da die Nachahmungsfähigkeit für einfache Bewegungen meistens lange erhalten bleibt und verbale Korrekturen (z. B. „bitte noch ein bisschen höher“) zugelassen sind. Dagegen liefert der Einsatz von Fragebogen in dieser Situation Aussagen unklarer Gültigkeit.

**2**. Je nach Setting können einige Fragen aktuell irrelevant sein, sodass der Patient die Antwort nicht kennt (im SPADI z. B. die Frage, ob 5 kg getragen werden können).

**3**. Wie schwer es vielen geriatrischen Patienten fällt, sich in der Beantwortung auch kurzer Fragen festzulegen, zeigt sich im erforderlichen Zeitaufwand. Dieser war für die Fragen, die mit den Handpositionen des 8P-GRT korrespondieren, deutlich höher als für die Performance. Beim SPADI waren viele Probanden zusätzlich mit der numerischen Bewertung ihrer Schwierigkeiten überfordert.

**4**. Beim 8P-GRT handelt es sich um eine strikt seitengetrennte Erfassung, während unilaterale Funktionsdefizite beim Einsatz von Fragebogen leicht übersehen werden können, solange die Kompensation über die Gegenseite noch zum Erhalt der Fähigkeit ausreicht.

**5**. Umgekehrt kann die Angabe einer Beeinträchtigung von Aktivitäten, die mit dem Greifraum assoziiert sind, in Fragebogen andere Gründe als die aktive Gelenkbeweglichkeit haben. So wurde z. B. Schwindel als Ursache für Probleme beim Haarewaschen angegeben.

Zwar ist die Erfassung des Greifraums längst auch sensor-/videogestützt möglich [13], die erforderliche technische Ausrüstung ist jedoch erst in wenigen Kliniken verfügbar und die Durchführung einschließlich Vor- und Nachbereitung erheblich aufwendiger als der 8P-GRT. Damit käme eine solche Erfassung ebenso wie eine detaillierte Untersuchung der Bewegungsgrade aller Gelenke, die Funktionsdefizite aufweisen, nur im Rahmen eines vertiefenden Assessments infrage.

In der Literatur fand sich kein praktikabler Test, der das Konstrukt des Greifraums geriatrischer Patienten seitengetrennt erfasst, als Vergleichsstandard. Die oben angeführten Aspekte der Kompensation über die andere Seite (→ evtl. nicht beeinträchtigt laut Fragebogen, beeinträchtigt laut 8P-GRT) sowie die Überlagerung durch Multimorbidität (→ evtl. beeinträchtigt laut Fragebogen, nicht beeinträchtigt laut 8P-GRT) erklären hinreichend, warum die Korrelation im Sinne konvergenter Validität nicht höher als beobachtet ausfiel. Zwischen den Ergebnissen des 8P-GRT und einem Fragebogen, der die korrespondierenden Alltagstätigkeiten bewertet, war sie moderat bis hoch (−0,72), mit dem SPADI moderat (−0,60).

Der 8P-GRT erfasst das Einnehmen von 8 alltagsrelevanten Handpositionen mit einem gut einminütigen Zeitaufwand/Seite mit hoher Reliabilität. Ergotherapeuten des Krankenhaus Rotes Kreuz Lübeck – Geriatriezentrum nutzen den 8P-GRT zusätzlich zur Handkraftmessung und zum 20-C‑T im Rahmen des Aufnahmeassessments. Viele der so diagnostizierten alltagsrelevanten Defizite waren nicht während der ärztlichen Untersuchung aufgefallen oder spontan vom Patienten kommuniziert worden. Liegt unter Berücksichtigung der Gesamtsituation ein Therapiebedarf vor, wird die Befundung fokussiert ergänzt.

## Limitationen

Die binäre Punktvergabe vereinfacht die Bewertung, limitiert jedoch Erwartungen an die Änderungssensitivität. Um Aussagen bezüglich der Änderungssensitivität treffen zu können, wurden zu wenige Patienten, die sich in der Therapie einer den Greifraum reduzierenden Erkrankung befanden, eingeschlossen. Hierin liegt eine Limitation der Studie. Auch war die Doktorandin, die für die Erhebung des 8P-GRT zuständig war, nicht für die Ergebnisse der weiteren Tests verblindet. Die hohe Interrater-Reliabilität lässt jedoch keine Zweifel an der Objektivität aufkommen.

## Fazit für die Praxis

- Ein Assessment der Mobilität sollte verstärkt die oberen Extremitäten einbeziehen, denn deren Einsatz ist von entscheidender Wichtigkeit für die Selbstversorgung und die Lebensqualität.
- Performance-Tests bieten gerade bei geriatrischen Patienten Vorteile gegenüber dem ausschließlichen Einsatz von Fragen.
- Mit der Messung der Handkraft, dem 20-Cents-Test (20-C-T) und dem 8‑Punkte-Greifraum-Test (8P-GRT) stehen an geriatrischen Patienten validierte Instrumente zur Verfügung, die mit hoher Reliabilität eine seitengetrennte Beurteilung erlauben.
